# Characterization of acyl carrier protein and LytB in *Babesia bovis* apicoplast

**DOI:** 10.1016/j.molbiopara.2011.10.009

**Published:** 2012-02

**Authors:** Marina C. Caballero, Monica J. Pedroni, Guy H. Palmer, Carlos E. Suarez, Christine Davitt, Audrey O.T. Lau

**Affiliations:** a Program of Genomics, Department of Veterinary Microbiology and Pathology and Paul G. Allen School for Global Animal Health, College of Veterinary Medicine, Washington State University, Pullman, WA 99164-7040, USA; b Animal Disease Research Unit, Agricultural Research Service, United States Department of Agriculture, Pullman, WA 99164-6630, USA; c Franceschi Microscopy and Imaging Center and the School of Biological Sciences, Washington State University, Pullman, WA 99164-4210, USA

**Keywords:** Apicomplexan, Apicoplast, *Babesia bovis*, *Plasmodium falciparum*, FASII, MEP, ACP, LytB

## Abstract

The apicoplast is a highly specialized organelle that mediates required functions in the growth and replication of apicomplexan parasites. Despite structural conservation of the apicoplast among different parasite genera and species, there are also critical differences in the metabolic requirements of different parasites and at different stages of the life cycle. To specifically compare apicoplast pathways between parasites that have both common and unique stages, we characterized the apicoplast in *Babesia bovis*, which has only intraerythrocytic asexual stages in the mammalian host, and compared it to that of *Plasmodium falciparum*, which has both asexual intraerythrocytic and hepatic stages. Specifically focusing on the type II fatty acid (FASII) and isoprenoid (MEP) biosynthesis pathways, we searched for pathway components and retention of active sites within the genome, localized key components [acyl carrier protein (ACP) and 4-hydroxy-3-methylbut-2-enyl diphosphate reductase (LytB)] to the apicoplast, and demonstrated that the N-terminal bipartite signals of both proteins are required and sufficient for trafficking to the apicoplast lumen. Using specific pharmacologic inhibition, we demonstrated that MEP biosynthesis may be disrupted and its presence is required for intraerythrocytic growth of *B. bovis* asexual stages, consistent with the genomic pathway analysis and with its requirement in the asexual erythrocytic stages of *P. falciparum*. In contrast, FASII biosynthesis may or may not be present and specific drug targets did not have any inhibitory effect to *B. bovis* intraerythrocytic growth, which is consistent with the lack of requirement for *P. falciparum* intraerythrocytic growth. However, genomic analysis revealed the loss of FASII pathway components in *B. bovis* whereas the pathway is intact for *P. falciparum* but regulated to be expressed when needed (hepatic stages) and silent when not (intraerythrocytic stages). The results indicate specialized molding of apicoplast biosynthetic pathways to meet the requirements of individual apicomplexan parasites and their unique intracellular niches.

## Graphical abstract

**Graphical abstract:**

Investigation of type II fatty acid and isoprenoid biosyntheses in Babesia resulted in the identification of two major components within the apicoplastic lumen.

**Figure:**
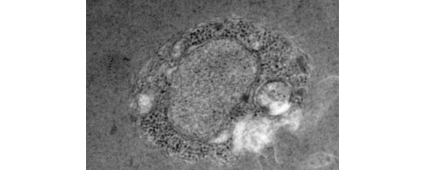

**Highlights:**

► This study illustrates a four membrane babesid apicoplast. ► *Babesia bovis* apicoplast resides adjacent to the nucleus. ► Acyl carrier protein and LytB are transcribed and translated in *Babesia bovis*. ► Isoprenoid biosynthesis likely exists in *Babesia bovis*. ► Type II fatty acid biosynthesis may not be present in *Babesia bovis*.

## 1 Introduction

The existence of a multi-membranous apicoplast within the phylum Apicomplexa was first observed in the 1960s and subsequently identified as a promising chemotherapeutic target due to its eubacterial and plant-like properties [1,2]. The apicoplast is the result of two separate endosymbioses with the engulfment of a cyanobacterium and an alga during the primary and secondary endosymbioses, respectively [3–5]. Within this organelle, two apicoplastic pathways, FASII and non-mevalonate 1-deoxy-d-xylulose-5-phosphate (DOXP) isoprenoid (MEP) biosyntheses, were identified and extensively investigated. Products from the FASII pathway participate in energy production and cell structure maintenance. Among the major components of the FASII pathway, acyl carrier protein (ACP) functions by holding the growing fatty acid chain covalently via its phosphopantetheine prosthetic group [6]. Thus, ACP is often used as a positive marker of apicoplast luminal protein for *Plasmodium* and *Toxoplasma* [7,8]. However, ACP is also found in mitochondria in plants and organisms such as *Neurospora* sp. as it forms part of the respiratory chain complex of NADH:ubuquinone oxidoreductase [9,10]. Thus, ACP can have dual functions. MEP biosynthesis is utilized exclusively by bacteria, Apicomplexan parasites, and plants as an alternative to the classic cytosolic mevalonate-dependant (MAD) mechanism, to produce isoprenoid precursors such as isopentenyl pyrophosphate (IPP) and dimethylallyl pyrophosphate (DMAPP) [11] which are involved in protein prenylation, cell membrane maintenance, protein anchoring and N′-glycosylation [12]. In this pathway, 4-hydroxy-3-methylbut-2-enyl diphosphate reductase (LytB, also known as IspH), catalyzes the final step of IPP and DMAPP production [13].

Characterization of FASII and MEP biosyntheses, mostly performed in *Plasmodium* and *Toxoplasma*, illustrates the crucial role in parasite survival [7,14]. Recent data reveal that not all apicoplasts are the same. Yu et al. and Vaughn et al. reported that FASII pathway is crucial only during the latter half of the liver stages in *Plasmodium falciparum* as it is not necessary during the mosquito and asexual erythrocytic stages [14,15] while *Toxoplasma* and *Eimeria* utilize both FASI and II for fatty acid production in the cytoplasm and apicoplast, respectively [16,17]. The *in silico* prediction of the lack of a FASII pathway in *Babesia bovis* [18], which lacks a liver stage but shares with *P. falciparum* asexual stage growth and replication within mature erythrocytes, further raises the question as to how ancestral apicoplast pathways have been molded to meet the specific needs of parasite species.

In the present study, we used four linked approaches to identify and characterize the apicoplast and two participants in FASII and MEP pathways in *B. bovis*: (i) visualization of the *B. bovis* four membrane apicoplast in the asexual stage; (ii) identification and localization of ACP and LytB; (iii) testing the role of the bipartite targeting signals in trafficking to the apicoplast lumen; and (iv) testing the sensitivity of the FASII and MEP pathways to specific pharmacologic inhibition. The results are presented and discussed in the context of these apicoplast pathways among Apicomplexan parasites, with specific comparison between *Plasmodium* and *Babesia*, two genera of parasite that infect erythrocytes as asexual stages.

## 2 Materials and methods

### 2.1 Parasite strain and in vitro cultivation of *B. bovis*

The Mo7 biological clone of *B. bovis* was derived by limited dilution of a Mexico strain as previously described [19,20]. Parasites were grown in long-term microaerophilous stationary-phase culture [21,20] followed by expansion cultures [22] using previously described techniques.

### 2.2 RNA isolation and cDNA generation

Total RNA was isolated from *B. bovis* cultures using TRIzol (Invitrogen), treated with RNase inhibitor (Roche) and RNase-free DNase (Turbo DNA-free from Ambion) for 30 min at 37 °C. RNA was reverse transcribed with RETROScript kit (Ambion) using oligo-dT primers, according to the manufacturer's instructions for a 2-step RT-PCR.

### 2.3 Identification and cloning of *B. bovis* acp and lytB genes

*B. bovis* acyl carrier protein gene (*acp*) specific primers were designed based on a sequence extracted from accession number NW_001820857. Full length *Bbacp* forward and reverse primers were 5′-ATG AAC GTT GTA TTC CGT ATG CTT AAC-3′ and 5′-TCA GAT GTC TTG TCG ACG TTC TAG CTT-3′, respectively. *Acp* was amplified from *B. bovis* cDNA using SuperTaq Polymerase (Ambion). PCR conditions were 95 °C for 3 min for 1 cycle followed by 94 °C for 30 s, 55 °C for 30 s, 72 °C for 2 min for a total of 30 cycles, and a final elongation step at 72 °C for 5 min. Amplified PCR product was visualized by electrophoresis and subsequently cloned into a pCR^®^4-TOPO^®^ vector (Invitrogen), following the manufacturer's protocol. Individual clones were selected, sequenced and analyzed (MacVector vers.11.1). *LytB* specific primers were designed based on a sequence extracted from accession number NW_001820857. Full length *lytB* forward and reverse primers were 5′-ATG CTA AAA TTA TTA TTT TTA ATA CTG-3′ and 5′-TTA TGT GCT GAC ACC CGG GC-3′, respectively. *LytB* was amplified from *B. bovis* cDNA cloned and sequenced using the same conditions as described above for *acp*.

### 2.4 5′ and 3′ Rapid Amplification of cDNA Ends for acp (RACE)

Total RNA was used as the template to generate 5′ RACE-Ready cDNAs by following the manufacturer's instructions (SMARTer RACE cDNA amplification kit, Clontech). Gene-specific primers with a Tm higher than 65 °C were designed. *Acp* RACE forward primer was 5′-ATG CTT AAC CCA TTG GGT ATT GCC TTT GTA GTC ATT CTA GCG C-3′. PCR was performed using Advantage 2 PCR kit (Clontech). Cycling conditions were: 94 °C for 30 s and 73 °C for 3 min for a total of 5 cycles; 94 °C for 30 s, 68 °C for 30 s and 72 °C for 3 min for 25 cycles, followed by 15 cycles of 94 °C for 30 s, 50 °C for 30 s, 72 °C for 2 min, and a final elongation step at 72 °C for 10 min. Amplified PCR products were confirmed by electrophoresis and corresponding bands were excised from the gel, purified using QIAquick Gel Extraction Kit (Qiagen) and subsequently sequenced.

### 2.5 Expression of recombinant mature *Babesia bovis* ACP and LytB

**Supplementary Fig. S1:**
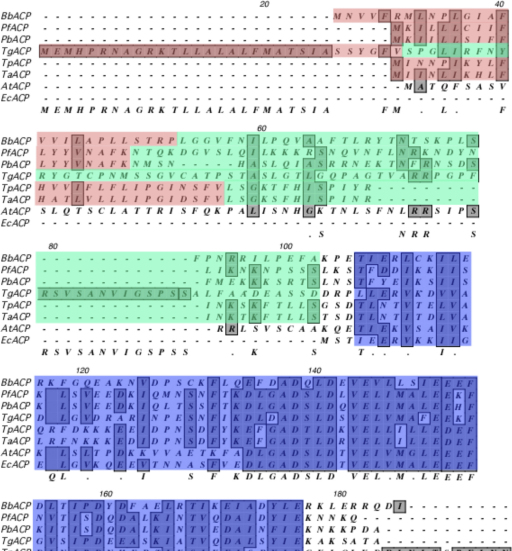
Deduced amino acid sequence alignment between eight acyl carrier protein (ACP) orthologues. Mature proteins containing the signature acyl carrier domain are shaded in blue while putative signal and transit peptides are shaded in red and green, respectively. Bb, *Babesia bovis*; Pf, *Plasmodium falciparum*; Pb, *Plasmodium berghei*; Tg, *Toxoplasma gondii*; Tp, *Theileria parva*; Ta, *Theileria annulata*; At, *Arabidopsis thaliana* and Ec, *Escherichia coli*. Both At and Ec do not contain putative signal and transit peptides.

Determination of the acyl carrier protein domain of ACP was based on consensus amino acid alignment between ACP orthologues (Fig. S1). Expression of the recombinant/mature ACP (rmACP_68–148_) protein, which is encoded by *acp*_202–444_, was carried out with the following forward and reverse primers, 5′-ACT ATA GAG CGC CTT TGT AAG ATT C-3′, and 5′-GAT GTC TTG TCG ACG TTC TAG CTT AC-3′. *Acp*_202–444_ gene was PCR-amplified from one of the previously generated clones, using the same PCR conditions described above. PCR product was cloned into a pTrcHis TOPO^®^ TA Expression vector (Invitrogen) and positive clones were confirmed by sequencing. Expression of the His-tagged rmACP_68–148_ was induced with a final concentration of 1 mM IPTG and visualized on 4–20% Tris–HCl SDS-PAGE electrophoresis system (Bio-Rad) stained with Coomassie Brilliant Blue R-250. Purification of rmACP_68–148_ was carried out using ProBond™ Purification System (Invitrogen) under denaturing condition, followed by electro-elution of gel-imbedded rmACP_68–148_. Briefly, after column purification of rmACP_68–148_, fractions were pooled and electrophoresed and the predicted sized band was excised and electro-eluted. The resulting electro-eluted protein was dialyzed to remove residual urea. SDS was also removed using SDS-Out precipitation kit (Pierce). Concentration of rmACP_68–148_ was determined using Micro BCA Protein Assay Reagent Kit (Pierce) with bovine serum albumin as a standard. Purified rmACP_68–148_ was detected by western blot using rabbit anti-His antibody (Santa Cruz Biotechnology). Antibody production of rmACP_68–148_ was subsequently generated (Pacific Immunology Corp., CA), affinity-purified using HiTrap™ Protein A HP Columns (GE Healthcare) and validated by western blot analysis using *B. bovis* infected erythrocytes, uninfected erythrocytes and recombinant ACP. Western blot analysis using pre-immune serum was also performed to ensure specificity of the immune serum.

**Supplementary Fig. S2:**
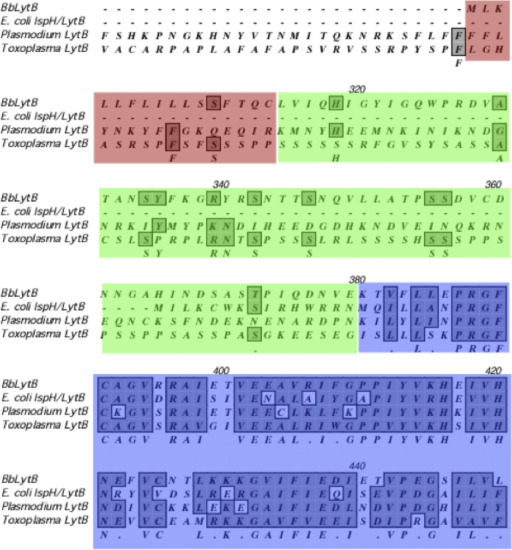
Deduced amino acid sequence alignment between four 4-hydroxy-3-methylbut-2-enyl diphosphate reductase (LytB/IspH) orthologues. Mature proteins containing the signature LytB active domain are shaded in blue while putative signal and transit peptides are shaded in red and green, respectively. Bb, *Babesia bovis*; *E. coli* does not contain putative signal and transit peptides. The full length LytB is not depicted in the alignment, only the amino terminus of the active domain is shown.

Determination of the active enzymatic domain of LytB was based on consensus amino acid alignment between four LytB orthologues (Fig. S2). Expression of the recombinant/mature LytB (rmLytB_82–419_) protein, which is encoded by *lytB*_246–1257_, was carried out with the following forward and reverse primers, 5′-AAG ACG GTG TTT TTA TTA GAG CCA CGA GGC-3′, and 5′-TGT GCT GAC ACC CGG GCT-3′. *LytB*_246–1257_ gene was PCR-amplified from one of the previously generated clones, using the same PCR conditions described above. PCR product was cloned into a pTrcHis TOPO^®^ TA Expression vector (Invitrogen) and selected clones were confirmed by sequencing. The His-tagged rmLytB_82–419_ was expressed and purified using the same protocol as described for rmACP_68–148_. Antibody production of rmLytB_82–419_ was subsequently generated (Pacific Immunology Corp., CA), affinity-purified using HiTrap™ Protein A HP Columns (GE Healthcare) and validated for specificity as applied to anti-ACP described above.

### 2.6 Immunofluorescent assay

*B. bovis* culture with 4% parasitized erythrocytes was incubated for 30 min at 37 °C with media containing 400 nM MitoTracker Orange CMTMRos (Invitrogen) for mitochondrial labeling. Cells were washed with fresh media and then centrifuged at 500 × *g* for 1 min. The infected erythrocyte pellet was mixed in a 1:10 ratio with 3% BSA/PBS and thin smears were subsequently prepared. Slides were covered with a 2% paraformaldehyde solution at pH 7.4, fixed for 5 min, and rinsed in 1× PBS, permeabilized with 0.2% Triton X-100/PBS for 10 min. All steps were performed in a wet chamber at room temperature unless noted. They were then washed twice with 1× PBS and blocked for 30 min at 37 °C with 3% BSA in PBS. Slides were then incubated with affinity purified anti-ACP rabbit IgG antibody at a 1:40 dilution for 1 h, washed twice with 1× PBS, and incubated with Alexa-Fluor^®^ 647 goat anti-rabbit at a 1:100 dilution for 1 h (Invitrogen). For the detection of native LytB, dilution of the affinity-purified anti-LytB IgG antibody was at 1:50. Controls include pre-immune serum at 1:30 dilution with the appropriate secondary antibodies, secondary antibodies only and uninfected erythrocytes.

For the detection of GFP-BSD reporter protein in the transfection experiments, anti-GFP antibody conjugated to Alexa Fluor 488 (Invitrogen) at 1:500 dilution was used along with anti-ACP (1:40) or anti-LytB (1:50) antibody and incubation was 30 min. After three washes with 1× PBS, coverslips were mounted on the slides using Vecta Shield mounting medium containing DAPI (Vecta Laboratories Inc.). Images were collected using a Zeiss LSM 510 META confocal laser scanning microscope equipped with 200 Axiovert inverted microscope using a C-APO 63X/1.2W.

### 2.7 Transient transfection of *B. bovis* infected erythrocytes

Fig. 3 is the schematic diagram of the plasmids used. Green fluorescent protein-blasticidin S deaminase (*gfp-bsd*) fusion gene was amplified from plasmid pTracer-CMV/bsd (Cat. No. V883-20, Invitrogen) using GFP *EcoRI* F (5′-CGA GGA ATT CAT GGC CTC CAA AGG AGA AG-3′) and BSD *EcoRI* R (5′-CTA TGA ATT CGC CCT CCC ACA CAT AAC CAG-3′) primers. The PCR product was then digested with *EcoRI* and cloned into plasmid p4-35-*ef-luc*, replacing the luciferace (*luc*) gene [23]. The resulting plasmid was designated p4-35-*gfp-bsd* (Fig. 3A). The predicted N-terminal bipartite apicoplast leading sequence (signal plus transit peptides, 1–60 amino acids (aa)) of ACP was amplified from *B. bovis* cDNA using primers ACP signal pept (1–22 aa) *EcoRI* F (5′-CTG AGA ATT CAT GAA CGT TGT ATT CCG TAT GCT TAA CCC A-3′) and ACP transit pept (23–60 aa) *BglII* R (5′-GAG AGA TCT CTC AGG TTT GGC-3′). In addition, a modified *gfp-bsd* fusion gene was amplified from plasmid pTracer-CMV/bsd this time using the above reverse primer, Bsd *EcoRI* R, and GFP-*BglII*-noATG F primer (5′-CGA AGA TCT GCC TCC AAA GGA-3′). Both amplicons, no ATG_*gfp-bsd* and SP + TP_ACP_ were digested with *BglII* and ligated to generate the SP + TP_*acp*_-*gfp-bsd* fusion gene, which was subsequently digested with *EcoRI* and cloned into the linearized, dephosphorylated p4-35-*ef* plasmid. The resulting plasmid was designated p4-35-SP + TP_*acp*_-*gfp-bsd* (Fig. 3B). Similar plasmid constructs were used to generate negative controls, p4-35-SP_*acp*_-*gfp-bsd* and p4-35-TP_*acp*_-*gfp-bsd* (Fig. 3C and D). All plasmids were purified using the Qiagen endotoxin-free maxiprep kit (Qiagen).

The predicted N-terminal bipartite apicoplast leading sequence (signal plus transit peptides) of LytB was amplified from *B. bovis* cDNA using primers LytB signal pept *EcoRI* F (5′-GGG CGA ATT CAT GCT AAA ATT ATT ATT TTT AAT ACT GCT TTC-3′) and LytB transit pept *BglII* R (5′-CGG CAG ATC TCT CTA CAT TAT CCT GTA TTG GTG-3′) and the *gfp-bsd* fusion gene was amplified as previously described. Both amplicons were digested with *BglII* and ligated to generate the SP + TP_*lytB*_-*gfp-bsd* fusion gene and subsequently cloned into p4-35-*ef* plasmid. The resulting plasmid was designated p4-35-SP + TP_*lytB*_-*gfp-bsd* (Fig. 3B). In the same fashion, plasmids p4-35-SP_*lytB*_-*gfp-bsd* and p4-35-TP_*lytB*_-*gfp-bsd* were also generated which code independently for the predicted N-terminal signal peptide (SP_*lytB*_) and transit peptide (TP_*lytB*_) of *B. bovis* LytB fused to *gfp-bsd*, respectively (Fig. 3C and D). Primers used to amplify SP_*lytB*_ were: LytB signal pept *EcoRI* F (described above) and LytB signal pept *BglII* R (5′-GCC CAG ATC TTA GAC ACT GTG TAA ATG A-3′) and primers used to amplify TP_*lytB*_ were: LytB transit pept *EcoRI*-ATG F (5′-GGC CGA ATT CAT GGT TAT CCA ACA TAT AGG TTA C-3′) and LytB transit pept *BglII* R (described above). All plasmids were purified as described above.

Electroporation of *B. bovis*-infected erythrocytes was performed as described by [23] in a Gene Pulser II apparatus (Bio-Rad) using 0.2 cm cuvettes containing 25 μl filter sterilized cytomix buffer (120 mM KCl, 0.15 mM CaCl_2_, 10 mM K_2_HPO_4_/KH_2_PO_4_ pH 7.6, 25 mM HEPES pH 7.6, 2 mM EGTA, 5 mM MgCl_2_, final pH 7.6) plus 100 μg of the corresponding plasmids and 75 μl of washed *B. bovis*-infected erythrocytes to a final volume of 100 μl. Control culture containing mock-transfected *B. bovis*-infected erythrocytes was included. Following electroporation, infected erythrocytes were cultured in 24-well plates as described above. The percentage of parasitized erythrocytes (PPE) was estimated daily by microscopic counting of smears stained with Diff-Quick^®^ (Dade Behring). Successfully transfected *B. bovis* were validated using PCR, RT-PCR, and western blot analysis (data not shown). Localization of GFP-BSD was also determined using immunofluorescent assay 4 h post-transfection as described in Section 2.7.

### 2.8 Electron microscopy

In order to visualize the apicoplast of *B. bovis*, infected erythrocytes were cultured to reach a PPE of ∼25%, as described by Levy and Ristic [21]. Infected erythrocytes were isolated from the culture by centrifugation at 350 × *g* for 10 min at 4 °C and then fixed with 2.5% glutaraldehyde in 0.1 M cacodylate buffer with 0.2 M sucrose for 2 h at RT without washing. Following 2× rinses with 0.1 M cacodylate buffer, samples were post-fixed in 2% osmium tetroxide in 0.1 M cacodylate buffer at 4 °C overnight, rinsed with water and placed in 1% aqueous tannic acid for 1 h. Samples were dehydrated in a graded ethanol series (30–100% 3×) infiltrated with acetone and embedded in Spurr's resin. Ultra-thin sections were stained with 2% uranyl acetate and Reynolds lead, and observed using a Philips CM 200 or JEOL 1200EX JEM transmission electron microscope (TEM).

### 2.9 In vitro targeted drug inhibition assay

Drug stock solutions of thiolactomycin (Sigma–Aldrich) and triclosan (Fluka) were prepared in tissue-culture grade dimethyl sulfoxide (DMSO). Fosmidomycin (Invitrogen) and Imidocarb diproprionate (ID) (Imizol^®^ Schering-Plough Animal Health) stocks were prepared in sterile water. Drug stocks were diluted daily to 1 μM, 10 μM, and 100 μM except ID which was diluted to a final concentration of 20 μM using culture media. All drugs were stored in the dark at 4 °C. The final DMSO concentration was 0.1%. Hydroethidine (HE) (Invitrogen) was dissolved in DMSO to a concentration of 10 μg/μl and stored at −20 °C [24]. Drug inhibition assay was conducted in microaerophilous stationary phase culture in 96-well plates. Each well contained 130 μl of culture at 10% hematocrit with a starting PPE of 0.5 as estimated by microscopic counting of smears stained with Diff-Quick^®^ (Dade Behring). Seventy-five percent of drug containing media was replaced at 24-h intervals. Negative controls included no treatment (NT) and 0.1% DMSO. PPE was enumerated by flow cytometry over a period of 72 h. Briefly, cultures were collected from triplicate wells at 24-h intervals and centrifuged at 0.4 × *g* for 5 min to remove media. The cell pellet was resuspended in 10× volume of 25 μg/μl HE and incubated at 37 °C for 20 min in the dark. Excess HE was washed from the cells by the addition of 1× PBS pH 7.4 followed by centrifugation at 0.4 × *g* for 5 min. The cell pellet was resuspended in fresh 1× PBS pH 7.4. PPE was then measured by flow cytometry using a Becton Dickinson FACSort (BD Biosciences) at a flow rate of <2000 events/s with 50,000 events collected. Data was analyzed using FCS Express 3 software (De Novo Software).

## 3 Results/discussion

### 3.1 Visualization of *Babesia bovis* apicoplast

**Fig. 1:**
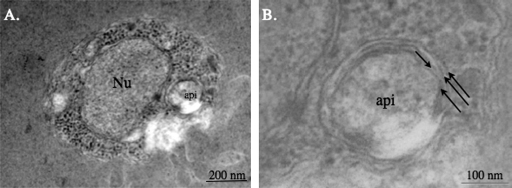
Visualization of *Babesia bovis* apicoplast using transmission electron microscopy. Nu, nucleus and api, apicoplast. (A) illustrates multi-membranous organelle, approximately 200 nm in diameter to be adjacent to the nucleus while (B) is a larger magnification of this organelle surrounded by four membranes, as pointed out by black arrows.

Complete genome sequencing of *B. bovis* revealed an apicoplast genome [18]. To test whether the intraerythrocytic asexual stage contained a structurally identifiable apicoplast, we used transmission electron microscopy and identified a multi-membranous organelle of approximately 200 nm in diameter (Fig. 1A). There are four membranes surrounding this organelle (Fig. 1B), the same as observed for the apicoplast morphology such as in *Toxoplasma* [25,26], *Garnia* [27] and *Sarcocystis* [28]. The number of membranes surrounding apicoplasts remains somewhat controversial as Hopkins et al. reported that *Plasmodial* apicoplast only contains three membranes [29]. As a result of this discrepancy, questions regarding shared ancestry with dinoflagellates, crytomonads and heterokonts remains unconfirmed [1].

*B. bovis* apicoplast is located adjacent to the nucleus (Fig. 1A), consistent with potential exchange of molecules between these two compartments [30]. However, we were unable to confirm if the apicoplast reside adjacent to the mitochondrion as well [1], as observed in *Plasmodium*. Multiple attempts were made to localize both the mitochondrion and apicoplast in the same field but were unsuccessful. Thus, our electron microscopy data cannot confirm if *Babesia* mitochondrion is also adjacently positioned to the apicoplast.

### 3.2 Identification of participants for the *B. bovis* FASII and MEP pathways

The original analysis of the *B. bovis* genome failed to identify the presence of genes encoding proteins involved in FASII biosynthesis, including a gene encoding ACP [18]. However, BLASTP search of the *B. bovis* genome using a consensus ACP active domain complied from multiple amino acid alignments with seven ACP orthologues identified an *acp*-like gene with 46–85% identity at the deduced amino acid level (Fig. S1). This gene is contained within a larger sequence, BBOV_I004020, and was initially annotated as encoding only a nucleolar GTP-binding protein 2, missing the *acp*-like sequence. A 476 bp fragment of *acp* was amplified using gDNA. It is predicted to have a small 34 bp intron between nt 287 and 321 (data not shown). *In silico* analysis of the deduced amino acid sequence of *B. bovis* ACP using SignalP (www.cbs.dtu.dk/services/SignalP) predicted a cleavable signal peptide between amino acids (aa) 1 and 22. BLASTP was used to locate the active enzymatic domain which is predicted to initiate around residue 61. This leaves a peptide domain spanning from aa 23 to 60 which may contain a putative transit peptide (TP) (Fig. S1). This putative TP is hydrophobic with 10% of its amino acids being basic in nature. Using RNA isolated from infected erythrocytes, cDNA was generated followed by 5′ RACE amplification to identify the 5′ untranslated region (UTR) as well as the putative translational start site of ACP. We detected a 442 base pair (bp) *acp* transcript predicted to encode a protein of 18 kDa. 5′ RACE amplification identified a 50 bp 5′ UTR and two putative translation start sites separated by 15 bp, shown in Fig. S1. Similar BLASTP searches of the *B. bovis* genome using consensus sequences of active domains of the remaining FASII participants failed to identify their orthologues in *B. bovis*. ACP is currently the only FASII pathway participant that has been identified, and shown to be transcribed and translated in *Babesia*. A new GenBank accession no. for *acp* has since been assigned (JN114412). Since ACP is also involved in the respiratory oxidation in mitochondria, ACP detected in *B. bovis* may be mitochondria-associated. However, the presence of the putative signal and transit peptides identified at the amino terminus of the protein, characteristic of a bipartite signal for targeting to the apicoplast, strongly suggests that *B. bovis* ACP likely reside within the apicoplast.

In contrast, the *B. bovis* genome encodes all of the components of the MEP pathway, including LytB. Multiple amino acid sequence alignments of four LytB orthologues subsequently identified the putative reductase domain of *B. bovis* LytB (BBOV_III001660) (Fig. S2). *In silico* analysis using SignalP and BLASTP for the predictions of signal peptide and the initiation of the active enzymatic domain, respectively, identified those regions to be between aa 1 and 16, and around aa 72, respectively (Fig. S2). This leaves a putative transit peptide to reside between aa 17 and 71 which is hydrophobic and basic, similar to the TP of ACP. Amplification of *lytB*, which is a gene of 1369 bp with three introns, and its respective 1261 bp transcript, were successful and identical to the predicted sequences previously reported [18] (data not shown).

### 3.3 Localization of ACP and LytB

**Fig. 2:**
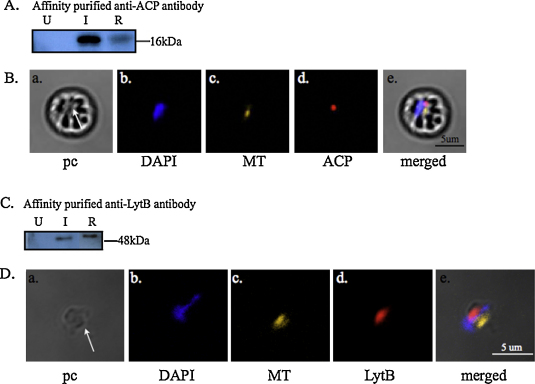
Western blot analysis was used to evaluate specificity of the antibodies raised against the mature active domain of ACP (A) and LytB (C). Affinity-purified anti-ACP or -LytB serum was tested on recombinant protein (R), uninfected (U) and infected (I) erythrocytes. Predicted sized proteins of ∼16 kDa and ∼48 kDa were detected for ACP and LytB, respectively. Localization of ACP (B) or LytB (D) was illustrated in an infected erythrocyte or free merozoite, respectively. White arrows indicate the location of the parasites. Specific antibody to ACP or LytB recognizes the native protein (d) to lie in a distinct compartment from those of the nucleus, stained with DAPI (b), and the mitochondrion, stained with Mitotracker (MT) (c). (a) and (e) The phase contrast (pc) and merged images, respectively.

To test whether ACP and LytB are localized to the apicoplast or the mitochondria, polyclonal antibodies were generated against recombinant proteins lacking both the putative signal and transit peptides. Western blot analyses were conducted using infected and uninfected erythrocytes on the immune sera to verify the specificity of the two antibodies (Fig. 2A and C). Predicted sized protein bands corresponding to the native ACP (∼16 kDa) and LytB (∼48 kDa) were detected in the infected erythrocytes only (Fig. 2A and C, lanes “I”). Preimmune sera were also tested and were unreactive to uninfected and infected erythrocytes as well as the recombinant proteins (data not shown). Mature luminal apicoplast proteins do not contain the signal and transit peptides. Evidence shows that the signal peptide is cleaved prior to apicoplast entry while the transit peptide is cleaved within the lumen [1,31]. For the immunofluorescent assay, the nucleus was labeled with DAPI (Fig. 2B-b and D-b) and the mitochondrion with Mitotracker-Orange (Fig. 2B-c and D-c). Our results demonstrate that ACP and LytB were localized to a discrete organelle (Fig. 2B-d and D-d). Merged images also suggest that this discrete organelle, presumably the apicoplast, may lie adjacent to the mitochondrion within the parasite (Fig. 2B-e and D-e), a result that our electron microscopy data were unable to confirm. These results also indicate that *B. bovis* does not contain a mitochondrial ACP.

### 3.4 N-terminal bipartite sequence is required for targeting proteins to the apicoplast lumen

**Fig. 3:**
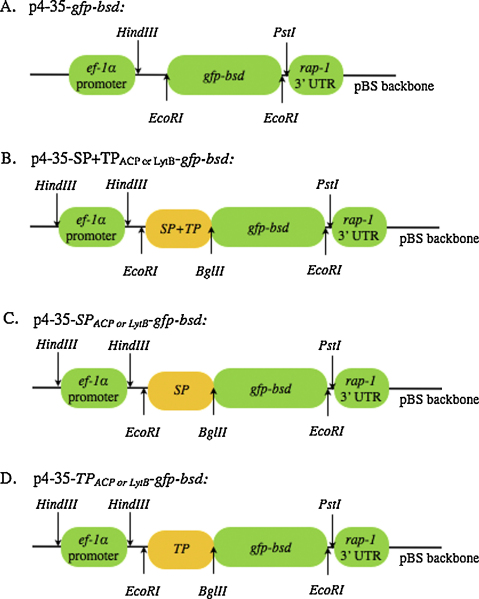
Schematic diagram of the constructs used in a series of *Babesia bovis* transient transfections. Controls include (A) plasmid with no bipartite sequence, (B) plasmid containing the bipartite sequence and the reporter cassette (GFP), (C) two plasmids, SP_ACP_ + GFP and SP_LytB_ + GFP and (D) two plasmids, TP_ACP_ + GFP and TP_LytB_ + GFP.

**Fig. 4:**
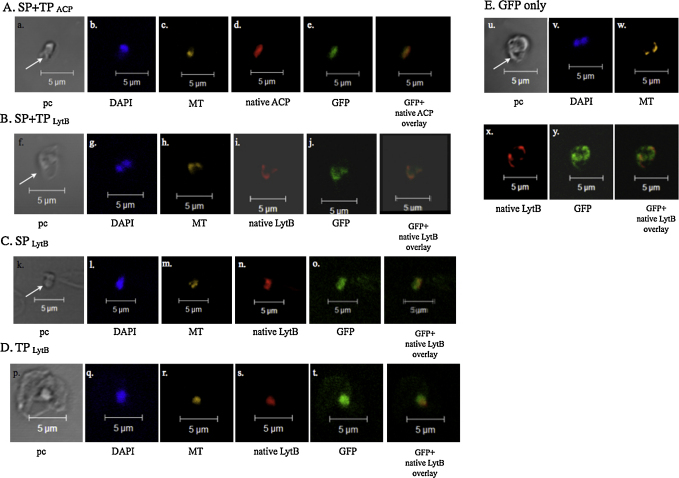
Immunofluorescent assay to detect *Babesia bovis* that were transiently transfected with (A) SP + TP_ACP_, (B) SP + TP_LytB_, (C) SP_LytB_, (D) TP_LytB_ or (E) GFP only. Detection of GFP was carried out using an anti-GFP antibody conjugated with Alexa Fluor 488. Using construct in (A) and (B), GFP localizes to a compartment as the native ACP (d, e) or LytB (i, j). In contrast, constructs containing only SP or TP, GFP fluorescence extends beyond the compartment where native ACP and LytB reside (C and D). pc, phase contrast; MT, mitotracker stained mitochondrion and DAPI stains nucleus. White arrows show free merozoites while (p) is a *B. bovis* infected erythrocyte. (E) represents a *B. bovis* transfected with a construct that lacks bipartite signal.

*In silico* sequence analysis indicated the presence of putative N-terminal signal (SP) and transit (TP) peptides (bipartite signal) for both ACP and LytB, a prerequisite shared by many nuclear-encoded proteins targeted to the apicoplast in *Plasmodium* and *Toxoplasma* [32]. To test if the bipartite signal is sufficient and required for correct trafficking of proteins to the apicoplast, *B. bovis* was transfected with SP + TP_ACP_-GFP-BSD or SP + TP_LytB_-GFP-BSD expression cassette (Fig. 3B). Controls included constructs that only contain SP_ACP_, SP_LytB_, TP_ACP_ or TP_LytB_. Mock transfection using p4-35-GFP-BSD was the negative control. Immunofluorescent assays performed 4 h post-transfection revealed the localization of GFP (Fig. 4A and B) in an organelle distinct from the nucleus and mitochondrion; the fluorescence staining of the GFP (Fig. 4A-e and B-j) was similar to that of the native ACP (Fig. 4A-d) and LytB (Fig. 4B-i), respectively, suggesting that both GFP and ACP or LytB reside in the same compartment. In contrast, neither SP nor TP alone, as illustrated by constructs SP_LytB_ + GFP or TP_LytB_ + GFP, directed GFP into the same compartment as the native LytB (Fig. 4C and D). Similar results were also obtained using constructs containing SP_ACP_ + GFP or TP_ACP_ + GFP (data not shown). Construct consisting only of the reporter cassette also did not direct GFP into the apicoplast as expected (Fig. 4E). These data collectively indicate that the complete bipartite signal (SP and TP) is necessary and sufficient for correct apicoplast trafficking.

### 3.5 Pharmacologic inhibition

Based on our genomic analyses of pathway composition and consequent expression data, we predicted that a functional MEP pathway exists within *B. bovis* while a functional FASII pathway does not. To determine the presence or absence of these two pathways, fosmidomycin was used to target MEP biosynthesis while thiolactomycin, triclosan and isoniazid were used to target FASII pathway. Fosmidomycin inhibits the action of DOXP reductoisomerase, blocking the conversion of DOXP to 2-*C*-methyl-d-erythritol 4-phosphate. Fosmidomycin also interacts indirectly with methylerythritol phosphate cytidyltransferase (IspD), an enzyme immediate downstream of DOXP reductoisomerase [33]. Thiolactomycin targets ketoacyl-ACP synthase (Fab B/F) in *P. falciparum* [34] while triclosan targets enoyl-acyl carrier protein reductase (Fab I) [35–37].

**Fig. 5:**
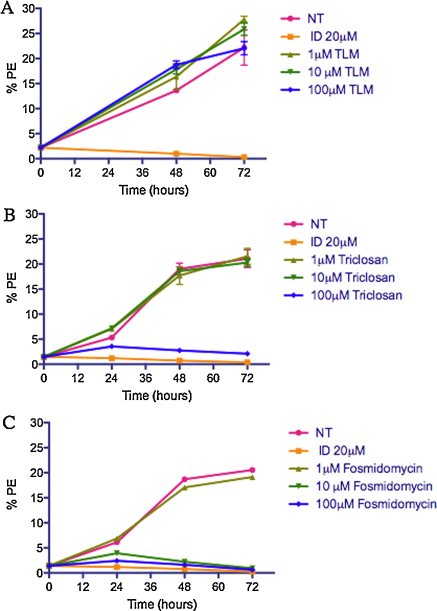
In vitro *Babesia bovis* growth inhibition assay using 1, 10 and 100 μM thiolactomycin (TLM) (A), triclosan (B) and fosmidomycin (C) over 72 h. Experiments were conducted in triplicates. Controls include no treatment (NT) and imidocarb diproprionate (ID) at a final concentration of 20 μM. %PE, percent parasitized erythrocytes.

Fosmidomycin was effective in the inhibition of *B. bovis* growth after 24 h at concentrations ≥10 μM (Fig. 5C). This effect has also been reported in *P. falciparum*, albeit at lower dosages [38]. The inhibitory effect of fosmidomycin on *B. bovis* indicates that its substrate(s) is/are present and further suggests that the MEP pathway is functional and required for intraerythrocytic growth of the asexual stages. In contrast, inhibition of the FASII pathway using thiolactomycin at concentrations between 1 μM and 100 μM showed no significant inhibition of *B. bovis* growth (Fig. 5A). Triclosan failed to inhibit *B. bovis* growth at 1 μM and 10 μM concentrations; while growth was inhibited with 100 μM triclosan (Fig. 5B), consistent with previous results [39]. However, despite earlier reports that claimed specific activity of triclosan, a recent study demonstrated nonspecific disruption of subcellular membrane structure at high concentrations [40]. Thus, the action of triclosan at 100 μM on *B. bovis* may not be Fab I-specific.

Together the genomic, expression, and pharmacologic data illustrate that significant differences may exist in apicoplast function among otherwise similar apicomplexan parasites. Specifically, a complete MEP pathway is likely to be present and functional in the asexual erythrocytic stages of *B. bovis*, similar to that of the asexual erythrocytic stages of *P. falciparum*. DOXP reductoisomerase is actively transcribed during the asexual blood stage (manuscript in prep.), further evidence supporting an active MEP pathway within *B. bovis*. The effectiveness of fosmidomycin may be due to the presence of new permeability pathways (NPP) found in erythrocytes. NPP alters the integrity of the red cell membranes [41,42]. This allows the trafficking of fosmidomycin to be effective against apicomplexans such as *Plasmodium* which resides within erythrocytes. In contrast, *Toxoplasma*, *Theileria* and *Eimeria* have all been reported to contain genes involved in MEP pathway and yet are non-responsive to fosmidomycin, even at 400 μМ [43–46]. Nair et al*.* recently shown that an apicoplast phosphate translocator (APT) located on the plasma membrane of the parasites is required to traffic fosmidomycin [46].

In contrast, a complete FASII pathway is probably lacking in *B. bovis* with only the presence of ACP. Consistent with this absence of the complete pathway, pharmacological inhibition within the specific inhibitor range had no effect on intraerythrocytic growth of *B. bovis* asexual stages. This lack of a functional requirement for the FASII pathway in asexual erythrocytic stages is supported by the lack of expression of FASII pathway participants (except ACP) and sensitivity to inhibition of this pathway in the erythrocytic stages of *P. falciparum* [14,15]. However, unlike *B. bovis* which only has the intraerythrocytic stages in the mammalian host, *P. falciparum* has a complete FASII pathway which is required for growth in the hepatic stages. These observations are consistent with specific molding of apicoplast pathways to meet the specific requirements of apicomplexan parasites, requirements which differ in a stage-specific/host cell-specific manner. *P. falciparum* retained the pathway for those stages where it was required but regulated expression in the asexual erythrocytic stages where the pathway is not needed. In the case of *Babesia*, *B. bovis* may have lost most of the pathway components as it adapts to asexual stage growth within mature erythrocytes. Thus, the apicoplast residing ACP may be the final footprint of a once functional FASII pathway which will soon be lost through evolution.
